# Cross-border COVID-19 spread amidst malaria re-emergence in Venezuela: a human rights analysis

**DOI:** 10.1186/s12992-020-00648-2

**Published:** 2020-12-17

**Authors:** Catalina Correa-Salazar, Joseph J. Amon

**Affiliations:** Department of Community Health and Prevention, Drexel University Dornsife School of Public Health, 3215 Market St, Philadelphia, PA 19104 USA

**Keywords:** Malaria, COVID-19, Re-emergence, Epidemic, Human rights, Violations, Venezuela

## Abstract

**Background:**

Since 2016 Venezuela has seen a collapse in its economy and public health infrastructure resulting in a humanitarian crisis and massive outward migration. With the emergence of the novel coronavirus SARS-CoV-2 at the end of 2019, the public health emergency within its borders and in neighboring countries has become more severe and as increasing numbers of Venezuelans migrants return home or get stuck along migratory routes, new risks are emerging in the region.

**Results:**

Despite clear state obligations to respect, protect and fulfil the rights to health and related economic, social, civil and political rights of its population, in Venezuela, co-occurring malaria and COVID-19 epidemics are propelled by a lack of public investment in health, weak governance, and violations of human rights, especially for certain underserved populations like indigenous groups. COVID-19 has put increased pressure on Venezuelan and regional actors and healthcare systems, as well as international public health agencies, to deal with a domestic and regional public health emergency.

**Conclusions:**

International aid and cooperation for Venezuela to deal with the re-emergence of malaria and the COVID-19 spread, including lifting US-enforced economic sanctions that limit Venezuela’s capacity to deal with this crisis, is critical to protecting rights and health in the country and region.

## Background

There is a growing recognition that the humanitarian crisis in Venezuela has unleashed a regional public health threat and a governance problem for Latin America [1]. Challenges related to the deteriorating Venezuelan public health system include the lack of: i) public health investment; ii) adequate health systems and disease control programs; iii) centralized epidemiologic information; and iv) protection of basic human rights for populations. Over the past few years, Venezuela has seen the unraveling of its government and health infrastructure. The censorship of epidemiologic surveillance data and persecution of health professionals, added to the scarcity of investment, has produced a dire situation for the population. Economic sanctions imposed by the United States and other nations have further restricted and undermined the health sectors capacity. In a context of intensifying governance and public health crisis and emergency, the re-emergence of once eliminated diseases like malaria and the massive outward migration of Venezuelans to neighboring countries has created major health systems challenges for the containment of cross-border disease spread, including the novel coronavirus, SARS-CoV-2. This has made the need for a global cooperation effort clear.

The aim of this paper is to discuss human rights-related factors of co-occurring malaria and COVID-19 epidemics in Venezuela, its border regions and neighboring countries to analyze potential ways forward. Bridging epidemiologic studies and human rights sources, we present two sets of analyses. The first addresses how the lack of governance and public health infrastructure have caused a health systems collapse that has permitted previously preventable diseases that were eliminated/controlled to re-emerge in Venezuela, particularly affecting historically underserved populations. This analysis includes a discussion of how massive migration caused by a concurrent economic and humanitarian crisis poses health systems threats to neighboring countries in Latin America [2–4]. The second analysis draws on human rights documents, standards and studies to discuss how the public health emergency results from and causes human rights violations within Venezuela, particularly to already underserved communities like indigenous groups [5]. These analyses provide an opportunity to understand how epidemiologic threats in cross-border contexts relate to human rights violations and the role and limits of global governance in response.

## COVID-19 in Venezuela and the region

As of mid-November 2020, Venezuela has reported more than 95,750 cases and 830 deaths of COVID-19 [6, 7]. It is likely these numbers are significant underestimates due to a lack of testing kits, supplies and the broken health infrastructure the country currently experiences [1]. Neighboring and near-by countries report vastly more cases. For example, Colombia has more than 1 million cases [8] and maintains a closed border policy with Venezuela and internal restrictions on social activities with hot spots-based quarantine measures. Perú has nearly as many cases and Brazil ranks third worldwide with more than 5,750,000 cases [9]. The situation is especially dire in the jungle-border regions that these Andean countries share, which are characterized by impoverished communities and limited health infrastructure [9].

The COVID-19 pandemic has exacerbated ongoing economic and political collapse in Venezuela [1], and compounds the challenges facing public health institutions from cuts in funding and corruption [10] resulting in shortages of medicines, medical personnel, health care supplies and basic infrastructure [11, 12]. Hospitals throughout Venezuela have reported water and electricity shortages that have severely hindered medical procedures and treatments [1, 13, 14]. In the last 5 years, Venezuela has seen both the re-emergence and an increased rate of infectious [3, 15], vector-borne [16, 17], vaccine-preventable [18] and neglected tropical diseases due to this crisis [19]; which has also affected neighboring countries and the region [15]. These health systems challenges to tackle diseases, are the same that the country now faces to deal with the spread of COVID-19.

The population health impact has been substantial. Venezuelans have reported losing 11 kgs. (24.2 pounds) on average of weight; maternal and child health indicators have worsened [20, 21], including maternal and child mortality [10]; and already vulnerable populations -mainly children, women, indigenous communities, the elderly and people suffering from pre-existing mental or physical health conditions and disabilities- have seen measures of quality of life worsen [22]. Furthermore, there is a lack of access to cancer medications and antiretroviral therapies for people living with HIV [1], with Venezuela being the only “middle-income country” country in the world were HIV therapies have been interrupted [23, 24]. These issues increase the population’s vulnerability to malaria and exacerbate risk factors for COVID-19 related to comorbidities, access to resources and care.

There are repeated water shortages in Venezuela [1, 12, 22], and it is estimated that nearly 80% of the population does not have continuous access to clean drinking water and basic sanitation [25]. Due to growing inflation, which is expected to reach 52,000% this last quarter of 2020 [26] and economic sanctions enforced by the United States and other countries [22], the public health situation continues to worsen as the government struggles with lack of public investment or funds to cover State debt [27]. Venezuela is now the poorest country in the Americas, and 96% of Venezuelan live in poverty [28]. Lack of sanitation conditions not only hinders vector control but also affects hygiene and cleaning practices necessary to stop the spread of COVID-19.

The collapse of Venezuela’s health infrastructure and deterioration of disease control programs has impacts on the region. The re-emergence of vector-borne and infectious diseases threatens neighboring countries as Venezuelan migrants and refugees that had fled the country are now returning as a result of quarantines in other countries and economic insecurity [29, 30]. Public health experts have started to warn about the potential syndemic [31] that could take place -if it has not already- due to convergence of increased numbers of dengue, malaria and measles [32] cases, the hindered immunization strategies due to the pandemic [33], the lack of HIV medications available [1], massive migration out and inward, and the immense human and economic costs that COVID-19 could bring to the region [1].

## Malaria re-emergence and COVID-19

Malaria is a vector-borne parasitic infection caused by several species of *Plasmodium* parasites and transmitted by *Anopheles* mosquitoes [2, 34]. Malaria can be prevented, and effectively treated with medicines included on the WHO’s list of “essential medicines” [34, 35]. In South America, malaria is most prevalent in the Amazon region (shared by Brazil, Perú, Ecuador, Bolivia, Guyana, French Guyana, Surinam, Venezuela and Colombia) [2]. Worldwide, in 2018, there were > 400,000 deaths of malaria, of which children account for roughly half [30].

Through a strategy of vector control and active case surveillance and response, malaria was eliminated in Venezuela in 1961 [36]. In Colombia, Ecuador and Brazil malaria control efforts are ongoing, with each country pursuing elimination [2]. Although Venezuela was the first WHO-certified country in Latin America to eliminate malaria in its most populated areas [36], the lack of vector control and surveillance in the last several years has resulted in the infection being reintroduced to formerly malaria-free areas, mainly in the Bolívar state in the South east corner of the country [37]. Cases of malaria from Venezuela have also been recorded in neighboring countries, accounting for 30% of new cases of malaria in the region in 2016 [36] and more than 50% of cases in 2017 [38, 39]. In 2018, the shared border region between Colombia, Brazil and Venezuela accounted for 80% of all malaria cases in the Americas; for which Venezuelan cases again represented more than half of all cases [38].

Malaria vulnerability is greater in populations that inhabit tropical and/or jungle areas in the region including among indigenous tribes; riverine, miners and fishing communities; as well as Afro communities relegated to isolated parts of Andean countries due to poverty and institutional racism [36]. In Venezuela, the risk of getting malaria escalates given the lack of vector control (fumigation, insecticide sprayed sleeping nets, indoor spraying and distribution of malaria prophylaxis medications) and barriers to access healthcare [38]. Additional factors that can be contributing to the epidemic relate to lack of testing, care and available medications for the ill, asymptomatic infections, peri-urban malaria, illegal gold mining-related malaria, malaria in pregnancy and possible under-detection; as well as lack of investment in public health infrastructure that supports preventive efforts and discontinuity of previously conducted elimination and eradication programs [2, 36]. While the Venezuelan government last published data on the incidence of malaria in the country in 2007, reports from international organizations and local clinics suggest high incidence [36]. Today, the country ranks first in malaria cases for the Americas, well over countries like Haiti, which have historically recorded high incidence of malaria [38].

The COVID-19 pandemic has made the situation in border zones even more challenging for malaria control efforts. Venezuelan migrants and refugees facing lockdowns, quarantine measures and even more scarce health resources as a result of the pandemic, could increase malaria transmission as they are forced to return to Venezuela and migrate again, often being held up in different cross border points for extended periods of time [29, 30]. Individuals with malaria needing care at home or in health care settings could also be at increased risk for COVID-19, and as Venezuelans seek to return to their country and/or get stuck along migratory routes, the potential importation and transport of coronavirus cases could be especially devastating given the country’s lack of infrastructure and public health system to care for patients [1, 12].

## COVID-19, health and human rights

The right to health is articulated in multiple human rights treaties including the Universal Declaration of Human Rights [UDHR] (Art.25) [40]; International Covenant of Economic, Social and Cultural Rights [ICESCR] (Art.12) [41]; Convention on the Rights of the Child [CRC](Art. 24) [42]; Convention on the Elimination of all forms of discrimination against women [CEDAW] (Art.12) [43]; United Nations Declaration on the Rights of Indigenous Peoples [UNDRIP] (Art. 24) [44]; Convention on the Rights of People with Disabilities [CRPD] (Art. 25) [45]; International Convention on the Elimination of All Forms of Racial Discrimination [CEAFRD] (Art. 5 (e.iv)) [46]. It is further articulated in General Comment 14 [47] of the UN Committee on Economic, Social and Cultural Rights which identifies social determinants of health as fundamental to the realization of the right to the highest attainable standard of health, including the right to clean water, the right to a healthy environment (like clean air, healthy environments and protection from toxic wastes), food, housing and jobs.

Realization of the right to health [41, 47] in Venezuela has been hindered by the government’s failure to take action to prevent vector-borne diseases like malaria, and now infectious diseases like COVID-19. In comparison with neighboring countries, Venezuelans are more likely to die from malaria and to arrive to the hospital with complication due to malnutrition [22] and health care in Venezuela has become progressively less available, accessibly, acceptable and of high quality. Health facilities commonly lack the necessary supplies and/or medicines to provide treatment, lack health personnel, and even basic provisions [11, 12, 22, 48]. This situation is worse for people living with disabilities, children, pregnant women and indigenous communities [4, 11].

Examining the actions, and inactions, of the Venezuelan government with respect to malaria and COVID-19, it is clear that they are failing to uphold their human rights obligations, both in terms of the overall population and in terms of populations subjects of special protection such as children, pregnant women, people living with disabilities, and indigenous communities [49]. This includes taking the necessary measures to reduce child morbidity and mortality, the prevention, treatment and control of epidemic, endemic, occupational and other diseases and the creation of conditions which would assure access to all medical service and medical attention in the event of sickness -all of which are aspects of the right to health as conceptualized in international human rights law as well domestic laws and Constitutional protection. The Venezuelan government’s failure to implement disease control measures, to collect and report epidemiological surveillance data and to cooperate with its neighboring countries to address the regional spread of infectious disease threats also entails a failure to uphold the WHO International Health Regulations [50]. Table 1 summarizes relevant human rights related to the re-emergence of malaria, COVID-19 and the government’s obligations from both international treaties and Constitutional protections.

**Table 1:** Human Rights violations and Venezuelan participation

Human Rights	Articles in Treaties, Conventions, Resolutions	Venezuelan Constitutional Inclusion of Rights violated
**Right to life**	CRC, Art. 6; CRPD, Art. 10; UNDIRP, Art. 7 (1); ICCPR, Art., 6	Title I: Fundamental Principles, Art 2. Title III: Of Human Rights, Protections and Responsibilities. Chapter 1: General Dispositions. Section2, Chapter 3: Of the Civil Rights, Art. 43.
**Right not to be discriminated against**	UDHR, 1948, Art 2; CEDAW, 4; CRC, 23; UNDRIP, 2, CRPD, 4; ICCPR, Art. 2; CMWF, Art. 7, 28	Title III: Of Human Rights, Protections and Responsibilities. Chapter 1: General Dispositions. Art 19 (no discrimination), Art. 20 (Free develpment of personality) and Art. 21 (right not to be discriminated against). Title III, Section 2: Of the Popular Referendum, Chapter 5: Of Social Rights and Families, Art. 88 (equality between men and women), Art. 89, 95 (no discrimination in work).
**Right to health**	UDHR, Art.25; ICESCR, 12; CRC, 24; CEDAW, 12; UNDRIP, 24; CRPD, 25; CEAFRD, Art. 5 (e.iv); CMWF, 28	Title III, Section 2: Of the Popular Referendum, Chapter 5: Of Social Rights and Families, Art. 83 (right to health), Art. 86 (Right to social security). Title IV: Of Public Power, Section 5, Chapter 4: Of Municipal Public Power, Art. 178 (5).
**Right to work**	UDHR, Art. 23 (1, 3); ICESCR, Arts. 6,7; CEAFRD, Art. 5 (e.i)	Title III, Section 2: Of the Popular Referendum, Chapter 5: Of Social Rights and Families, Art. 89 (right to work)
**Right to housing**	UDHR, 25; ICESCR, 11 (1); CEAFRD, Art. 5 (e.iii)	Title III, Section 2: Of the Popular Referendum, Chapter 5: Of Social Rights and Families, Art. 82.
**Right of special protection**^a^	For mothers: ISECSR, Art. 10 (2,3) Children: ICCPR, Art 24 Indigenous populations: UNDRIP, Art. 22	Title III, Section 2: Of the Popular Referendum, Chapter 5: Of Social Rights and Families, Art. 76: Focus on maternal and child health protection. Art. 78: Focus on children. Title III, Section 2:Chapter 8: Of the rights of indigenous peoples. Arts. 119–126.
**Right to information**	ICESCR, Art 12(a,b,c,d)	Title III: Of Human Rights, Protections and Responsibilities. Chapter 1: General Dispositions.Art. 28 (access to information). Title III, Section2, Chapter 3: Of the Civil Rights. Art. 58: Right to information for all. Focus on children. Title III: Of Human Rights, Protections and Responsibilities. Chapter 6: Of Cultural and Educational Rights, Art. 108 (State supported of communication networks, social media and cultural means of information).
**Right to a healthy environment**	Indigenous populations: UNDRIP, Art. 29 (1,2,3) Convention on the Rights of Mother Earth (2016)	Title III, Section 2: Of Popular Referendum, Chapter 7: Of Economic Rights, Art. 112 Title III, Section 2:Chapter 9: Of environmental rights. Arts. 127–129. Title IV: Of Public Power, Section 5, Chapter 4: Of Municipal Public Power, Art. 178 (4).

^a^Granted to groups that have been historically and/or socially discriminated against

### Case study: Venezuela’s indigenous communities

Bolívar State, with a high concentration of illegal gold mines, accounted for 74% of Venezuela’s malaria in 2016 - the highest incidence in the Americas [51]. Illegal gold mining affects ecosystems as it involves vast deforestation and contamination of food and water sources, which favors mosquito proliferation [36]. The increase in malaria incidence has been associated with these human activities and the massive migration to these areas of people looking for jobs [52]; but also to the conditions in which miners work, being overexposed to long periods outside and pressured to live in camps where living conditions lack sanitary standards of health and protection (complete walls, mosquito nets, roof protection) [36, 51]. This violates their rights to work and housing as understood in international human rights law (ICESC, Arts. 6,7; CEAFRD, Art. 5 (e.i)). The Venezuelan State has neglected to take effective measures that ensure the protection of the environment and the disposal of hazardous materials derived from mining, which greatly harms indigenous communities in the area [53], violating their right to a clean environment and the protection of its resources according to the Declaration on the Rights of Indigenous People and the Venezuelan Constitution [54]. Expansion of endemic transmission from Zulia to Mérida is also due to domestic massive migration patterns that spread the disease to areas of chronic under-investment in public health in the Southeast of the country [52].

Indigenous people have repeatedly denounced the exploitation and detrimental impact that mining has on their territories, bringing settlers into their land, increasing disease infection rates and exposing their communities, particularly women and children, to sexual and labor exploitation [55, 56]. COVID-19 aggravates this situation, which is compounded by the lack of efforts to prepare for or address the crisis, especially in the Amazon and Amazon-adjacent areas [9]. In addition to illegal mining, Bolívar state, has high levels of drug and human trafficking, sexual exploitation and illegal guerrilla activities involving training, children’s forced recruitment and labor exploitation under arbitrarily declared ‘war laws’ [5]. The scarcity of health infrastructure is also exacerbated by the lack of occupational health care arising from illegal labor [5]. Lack of information on malaria prevention and treatment for indigenous populations also contribute to poor health outcomes and represents failures of the government to uphold human rights obligations [17].

Indigenous communities were recognized by the Venezuelan government as part of the country in the 1999 Constitution (see Table 1) and Venezuela proclaimed itself a multicultural nation [54]. However, the government has historically protected private companies and investors that exploit natural resources in Amazon-adjacent and border territories, infringing indigenous people’s rights to land, resources and protection, and failing to uphold human rights obligations related to the right to a healthy environment. Under the guise of responding to the economic impact of the COVID-19 pandemic, the rights of indigenous communities in Venezuela could be further threatened, as has been the case in Brazil, where the country’s President, Jair Bolsonaro, has stripped the Amazon and the indigenous communities of constitutional protections that protect them, their land and the ecosystem [57, 58]. In Venezuela, mining companies operate under the protection of the army, which is suspected to take a part of the royalties in exchange for services provided [5]. Human rights and international organizations have documented how indigenous women are forced into prostitution by mining activities and further exposed to violence under territory disputes between armed illegal actors [55, 56]. These communities are often forced to flee in search of protection, which causes them to seek refuge outside of their ancestral land in neighboring countries, potentially exacerbating vulnerability to infection and the spread of disease.

## Conclusion

The Venezuelan State has neglected to take effective measures that ensure the protection of the environment, of groups of special protection and of the general population; and avoided confrontation with mining activities, illegal guerrillas and gangs that enforce armed control [5]. The United Nations Office of the High Commissioner for Human Rights declared in September 2020 that the government was complicit with censorship, repression of peaceful protests, forced disappearances, politically motivated detention and torture, extrajudicial executions and a compromised judiciary, with a system that fails to serve as a check on other State actors [59]. These rights violations can directly impact health, at an individual and population level [60].

The Venezuelan government’s rights obligations can be understood both in terms of its failure to implement what is required under international treaties and conventions (positive obligations), but also in the purposeful actions that it is *actively* taking to censor and suppress access to information on the spread of malaria and COVID (negative obligations) [51]. By allowing the public health system to collapse under lack of funding and protection, but also by permitting illegal activities under its jurisdiction like mining and guerrilla presence [5], the Venezuelan government is violating its own Constitution and is failing to ensure Venezuelans in general, and children’s, women’s, indigenous communities’ rights in particular, including the rights to life and health.

This failure to control malaria and prevent its re-emergence is caused by the lack of availability, quality, accessibility and acceptability of healthcare institutions, health personnel, health facilities, supplies and trustworthy public epidemiologic information. The government of Venezuela does not support the life, development, non-discrimination, work, housing, environment and highest attainable standard of health of its populations and the lack of implementation of malaria prevention strategies as defined by the WHO, the United Nations and its own domestic laws. The right to health, as stated in international conventions, the ICESCR and General Comment 14, incentivizes cooperation and support on the part of the international community to progressively achieve its full realization if necessary or required. Malaria re-emergence and its convergence with the COVID-19 pandemic, as presented here, should be dealt with internationally as it has the potential to impact other nation’s subjects of special protection and refers to a population that is currently undergoing a humanitarian crisis and a global pandemic.

## Ways forward

The necessary measures to protect human rights, particularly to ensure the right to health, must include cooperation with international funders and agencies to support public health infrastructure, medicines, supplies and personnel that have been turned down by the Venezuelan government in the past. Disclosure of public health surveillance data is paramount in order to assess and intervene syndemic situations relating to malaria and the COVID-19 pandemic. International organizations and local clinics on the ground have been critical in reporting incidence, trends and spread of malaria [36], and can once again contribute to assess, based on more realistic numbers, the COVID-19 situation within Venezuela without fear of being persecuted, censored and/or closed. Consequently, the right to information is central to protect the right to health in this context.

Ensuring the protection of the right to health and other human rights in Venezuela requires international agencies and non-governmental organizations to support efforts within Venezuela and in neighboring countries and border regions to expand the provision of healthcare and medicine distribution for Venezuelans, border dwellers and migrants/refugees in these areas; particularly for indigenous populations in the Amazon and Amazon-adjacent territories that are being disproportionally affected by COVID-19 and factors impacting human rights. Given the restrictions of information concerning malaria, expanded efforts are needed too to ensure that accurate estimates of COVID-19 cases are reported from the country. Protection of medical personnel, which has been heavily repressed in the past must be a goal of international cooperation and a focus on the extra-territorial principle of the responsibility to protect of the international community while dealing with COVID-19 control strategies in the area.

The continuity and commonality between the claims in human rights abuses denounced has increased international pressure since the crisis in Venezuela was first declared in 2016. International traction gained by the United Nations and different international human rights platforms [61, 62] and international cooperation agencies -including the UN Human Rights report on Venezuela to take immediate measures to halt and remedy grave rights violations in 2020 [63] has been useful to promote COVID-19 responses in Venezuela [64, 65]. The Pan American Health Organization, the WHO, UNHCR and OCHA have all been instrumental in coordinating important diplomatic actions to enforce the Right to Protect through support to neighboring nations, fund raising and supply distribution [64, 66, 67]. These actions, however, will be hindered if economic sanctions enforced by the United States are not lifted and Venezuela lacks economic capacity to maneuver. Infrastructure investments are needed in Venezuela to articulate coordinated responses both within and in tandem with the international community. The pictures, reports, research and interventions on the massive migratory flows leaving Venezuela (and now in transit or returning due to pandemic-associated measures in countries in the region) have created momentum to expose the suffering of Venezuelans and create awareness of the situation on international platforms.

The response of countries like Colombia, which has included Venezuelan migrants/refugees in its response to COVID-19 [68], will not be enough without a global support and action from Global North funding and cooperation, including U.S. agencies. Actions to promote change should focus on containment of vector-borne, vaccine-preventable, neglected diseases and human rights. Surveillance, monitoring and prevention of the development of the public health crises caused by human rights abuses and syndemic effects with infectious diseases can increase grave human rights violations and cause grave health effect for populations and generations to come.

## Data Availability

All materials cited in this publication and consulted research can be consulted in the cited references. We did not consult any data bases that are privately owned or inaccessible to the public. Data sharing not applicable to this article as no datasets were generated or analyzed during the current study.

## Abbreviations

UDHR: Universal Declaration of Human Rights
ICESCR: International Covenant of Economic, Social and Cultural Rights
CRC: Convention on the Rights of the Child
CEDAW: Convention on the Elimination of all forms of discrimination against women
UNDRIP: United Nations Declaration on the Rights of Indigenous Peoples
CRPD: Convention on the Rights of People with Disabilities
CEAFRD: International Convention on the Elimination of All Forms of Racial Discrimination
